# Myricanol Induces Apoptotic Cell Death and Anti-Tumor Activity in Non-Small Cell Lung Carcinoma *in Vivo*

**DOI:** 10.3390/ijms16022717

**Published:** 2015-01-26

**Authors:** Guanhai Dai, Yeling Tong, Xuan Chen, Zeming Ren, Xuhua Ying, Feng Yang, Kequn Chai

**Affiliations:** 1 Institute of Basic Medicine, Zhejiang Academy of Traditional Chinese Medicine, Hangzhou 310007, China; E-Mails: tongyeling@sina.com (Y.T.); chenxuan001564@163.com (X.C.); rchao007@163.com (Z.R.); 88082214@163.com (F.Y.); 2 Institute of Cancer Research, Zhejiang Academy of Traditional Chinese Medicine, Hangzhou 310007, China; E-Mail: xuhuaying668@sina.com; 3 Oncology Department, Tongde Hospital of Zhejiang Province, Hangzhou 310012, China

**Keywords:** myricanol, anticancer, A549 xenograft, immunohistochemistry, TUNEL assay, apoptosis

## Abstract

This study explored the inhibiting effect and mechanism of myricanol on lung adenocarcinoma A549 xenografts in nude mice. Forty nude mice with subcutaneous A549 xenografts were randomly divided into five groups: high-dose myricanol (40 mg/kg body weight) group; middle-dose myricanol (20 mg/kg body weight) group; low-dose myricanol (10 mg/kg body weight) group; polyethylene glycol 400 vehicle group (1 mL/kg); and tumor model group. Nude mice were sacrificed after 14 days of treatment and the tumor inhibition rate (TIR, %) was then calculated. The relative mRNA expression levels of Bax, Bcl-2, VEGF, HIF-1α, and survivin in the tumor tissues were determined by real-time PCR. TUNEL assay was applied to determine cellular apoptosis, while IHC test was performed to detect the protein expression levels of Bax, Bcl-2, VEGF, HIF-1α, and survivin. The TIR of the three myricanol-treated groups ranged from 14.9% to 38.5%. The IHC results showed that the protein expression of Bcl-2, VEGF, HIF-1α, and survivin were consistently downregulated, whereas that of Bax was upregulated after myricanol treatment. Myricanol also significantly upregulated the mRNA expression of Bax and downregulated that of Bcl-2, VEGF, HIF-1α, and survivin in a dose-dependent manner (*p* < 0.05 to 0.001). These results are consistent with those of IHC. The TUNEL assay results indicated that apoptotic-positive cells significantly increased in the myricanol-treated tumor tissues compared with the cells of the vehicle control group (*p* < 0.01 to 0.001). These data suggest that myricanol could significantly decelerate tumor growth *in vivo* by inducing apoptosis.

## 1. Introduction

Lung cancer is one of the most commonly diagnosed malignancies and leading causes of cancer-related deaths [1]. Furthermore, lung cancer is divided into small-cell lung cancer (SCLC) and non-small cell lung cancer (NSCLC). NSCLC accounts for 80% to 85% of all lung cancer cases; as such, the pathogenic mechanism of NSCLC should be understood [2]. Despite the availability of chemotherapy regimens, the mortality rate of NSCLC has not decreased [3]. Therefore, novel anticancer agents should be developed to improve pharmacological profiles and increase survival from NSCLC.

Myricanol is a bioactive agent extracted from *Myrica* bark [4,5,6]. This agent exhibits many biological activities, including reversal of Alzheimer’s disease [7], inhibition of nitric oxide production and inhibition of degranulation; it also has anti-inflammatory [8], anticancer [9], and anti-androgenic effects [10]. Inflammation is, in certain cases, evident at the earliest stages of neoplastic progression and demonstrably capable of fostering the development of incipient neoplasias into full-blown cancers. However, information regarding the anticancer mechanism of myricanol is limited. Therefore, this study was conducted to investigate the antitumor and apoptotic effects of myricanol *in vivo*.

In our previous work, we investigated the pro-apoptotic and antitumor effects of myricanol on many cancer cell lines, including HL-60 and HepG2. Myricanol can significantly inhibit the growth of A549 cells in a dose-dependent manner, decrease colony formation, and induce A549 cell apoptosis *in vitro*. Myricanol can upregulate the expressions of caspase-3, caspase-9, Bax, and p21 and downregulate the expression of Bcl-2 at mRNA and protein levels [9]. These changes have been associated with apoptosis.

In this study, the therapeutic effects of myricanol on the xenografts of athymic nude mice of human NSCLC tumor were investigated. The results showed that myricanol can suppress tumor growth *in vivo*. Furthermore, myricanol can significantly upregulate the mRNA expression of Bax and downregulate the mRNA expression of Bcl-2, vascular endothelial growth factor (VEGF), hypoxia-inducible factor (HIF)-1α, and survivin in a dose-dependent manner. These results are consistent with the immunohistochemical (IHC) findings. TUNEL assay results indicated that apoptotic-positive cells significantly increased in myricanol-treated tumor tissues. These data provided evidence regarding the therapeutic potential of myricanol as an anticancer drug in NSCLC.

## 2. Results and Discussion

### 2.1. Results

#### 2.1.1. Antitumor Effect of Myricanol on an A549 Cell Xenograft Model

**Figure 1:**
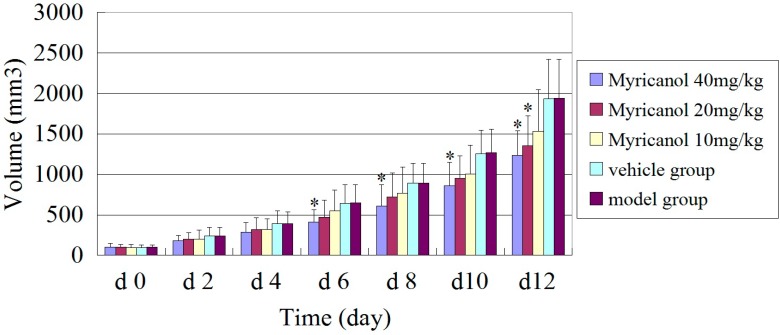
Growth curve of tumor volume. Tumor xenografts from A549 cells were established in athymic nude mice in the flanks and treated with either myricanol or PEG-400 (vehicle control) for 14 days consecutively. Tumor volume was measured with Vernier caliper and calculated. * Compared with the vehicle group, *p* < 0.05.

**Figure 2:**
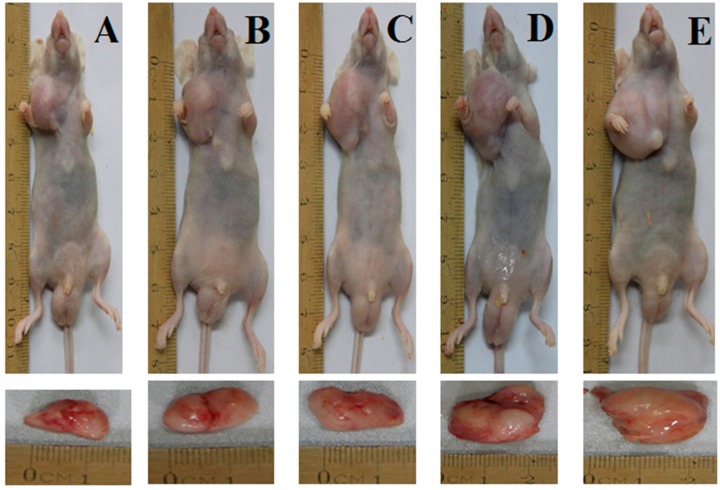
Antitumor effect of myricanol on A549 cells in nude mice. Tumor xenografts from A549 cells were established in athymic nude mice in the flanks and were treated with either myricanol or PEG-400 (vehicle control) for 14 days consecutively. (**A**) Myricanol with 40 mg/kg; (**B**) myricanol with 20 mg/kg; (**C**) myricanol with 10 mg/kg; (**D**) vehicle control group; and (**E**) tumor model group.

We used an A549 xenograft model to investigate the antitumor effect of myricanol on A549 cells *in vivo*. The tumor formation rate in nude mice was 100%. The standard (≈100 mm^3^) was observed after 10 days, and the volume of each tumor was measured by sliding calipers at 2 days interval. The periodic measurement of the tumor xenograft volume indicated that the tumor volume in nude mice decreased significantly in the highest concentration of the myricanol group (40 mg/kg body weight) compared with the vehicle group (*p* < 0.05) after 6 days of the experiment (Figure 1). The tumor volume also decreased significantly in the middle-dose myricanol (20 mg/kg body weight) compared with the vehicle group (*p* < 0.05) after 12 days of experiment. The myricanol-induced inhibitions of the A549 xenograft tumor volume in mice administered with myricanol at 40 and 20 mg/kg body weight concentrations were 39.4% and 25.5%, respectively. At the termination of the experiment, the weight of the tumor in each treatment group was significantly decreased in three different myricanol doses (40, 20, and 10 mg/kg body weight) compared with the vehicle and tumor model groups (*p* < 0.05, Figure 2). The TIRs of the three myricanol doses ranged from 14.9% to 38.5% (Table 1). The differences between the vehicle and model groups were not significant (*p* > 0.05). No animal death occurred during the experiment, and the body weight of the myricanol group did not significantly differ from that of the model group.

**Table 1:** Antitumor effect of myricanol on an A549 cell xenograft model (*n* = 8, $\bar{x}$ ± SD).

Group and dose	Body Weight (g)		Tumor Weight (g)	TIR (%)
	Begin	End		
Myricanol (40 mg/kg)	20.9 ± 1.43	24.9 ± 2.21	1.894 ± 0.555 *	38.5
Myricanol (20 mg/kg)	21.4 ± 1.81	24.8 ± 2.13	2.239 ± 0.782 *	27.3
Myricanol (10 mg/kg)	22.1 ± 1.92	24.4 ± 2.12	2.628 ± 1.021	14.7
Vehicle group	21.7 ± 1.15	25.0 ± 2.05	3.079 ± 0.834	0.81
Model group	21.5 ± 1.28	25.3 ± 1.95	3.104 ± 0.901	-

* Compared with the vehicle group *p* < 0.05.

#### 2.1.2. Immunohistochemistry Analysis of Bax, Bcl-2, VEGF, HIF-1α, and Survivin Expression 

**Figure 3:**
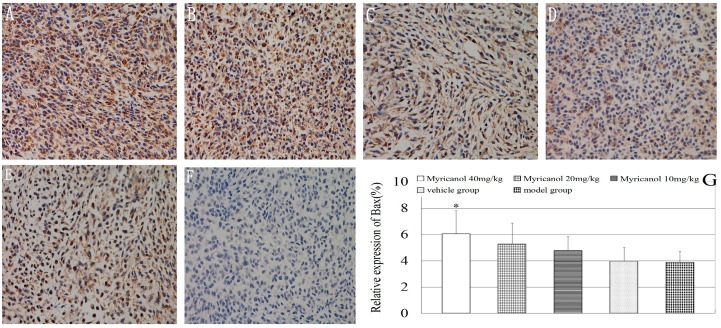
Immunohistochemical detection of BAX protein in A549 cells (magnification of 400×). The relative expression of Bax was determined by NIS-Elements D 3.2 image analysis system. (**A**) Myricanol with 40 mg/kg; (**B**) myricanol with 20 mg/kg; (**C**) myricanol with 10 mg/kg; (**D**) vehicle control group; (**E**) tumor model group; and (**F**) isotype control. The yellow and brown particles represent positive BAX expression; (**G**) Quantification of protein expressions in different groups by IHC. * Compared with the vehicle group, *p* < 0.05.

We examined tumor xenograft samples from each treatment group for expressions of Bax using IHC analysis to further determine the mechanisms involved in myricanol-mediated induction of the apoptosis of lung tumor cells *in vivo*. The relative expression of Bax in the tumor of nude mice in the highest myricanol dose group increased significantly compared with that in the vehicle group (*p* < 0.05, Figure 3). The relative expression level of Bax between the vehicle and model groups was not significant (*p* > 0.05). 

**Figure 4:**
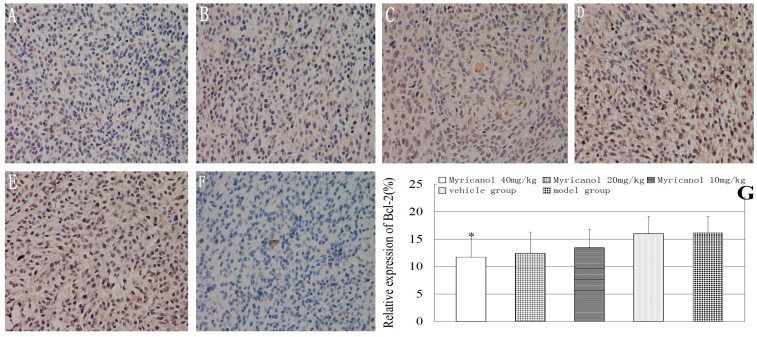
Immunohistochemical detection of Bcl-2 protein in A549 cells (magnification of 400×). The relative expression of Bcl-2 was determined by NIS-Elements D 3.2 image analysis system. (**A**) Myricanol with 40 mg/kg; (**B**) myricanol with 20 mg/kg; (**C**) myricanol with 10 mg/kg; (**D**) vehicle control group; (**E**) tumor model group; and (**F**) isotype control. The yellow and brown particles represent positive Bcl-2 expression; (**G**) Quantification of protein expressions in different groups by IHC. * Compared with the vehicle group, *p* < 0.05.

We examined tumor xenograft samples from each treatment group for Bcl-2 expression using IHC analysis. Bcl-2 presented strong immunoreactivity in the vehicle control and tumor model groups (Figure 4). After myricanol treatment, the highest dose of myricanol significantly suppressed Bcl-2 expression level compared with the vehicle group (*p* < 0.05). The relative expression of Bcl-2 between the vehicle and the model groups was not significant (*p* > 0.05).

**Figure 5:**
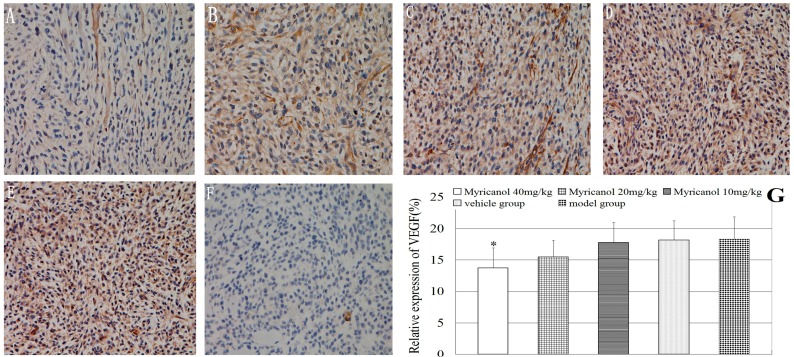
Immunohistochemical detection of VEGF protein in A549 cells (magnification of 400×). The relative expression of VEGF was determined by NIS-Elements D 3.2 image analysis system. (**A**) Myricanol with 40 mg/kg; (**B**) myricanol with 20 mg/kg; (**C**) myricanol with 10 mg/kg; (**D**) vehicle control group; (**E**) tumor model group; and (**F**) isotype control. The yellow and brown particles represent positive VEGF expression; (**G**) Quantification of protein expressions in different groups by IHC. * Compared with the vehicle group, *p* < 0.05.

We examined tumor xenograft samples from each treatment group for the expressions of VEGF using IHC analysis to further determine the antitumor effect of myricanol on A549 cells *in vivo*. The relative expressions of VEGF were significantly lower in the highest myricanol dose group than in the vehicle group (*p* < 0.05, Figure 5). Myricanol may inhibit the growth and angiogenesis of human lung adenocarcinoma by inhibiting VEGF expression. The relative expression of VEGF between the vehicle and the model groups was not significant (*p* > 0.05).

**Figure 6:**
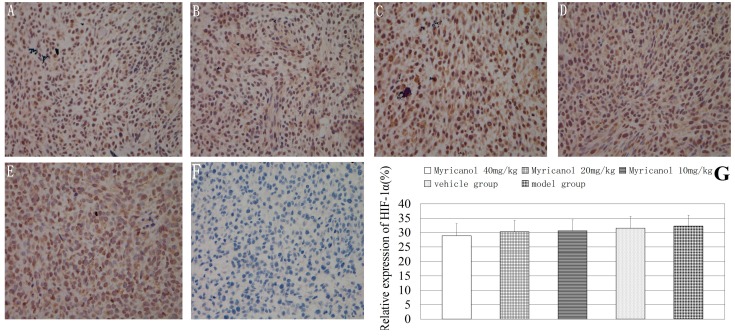
Immunohistochemical detection of HIF-1α protein in A549 cells (magnification of 400×). The relative expression of HIF-1α was determined by NIS-Elements D 3.2 image analysis system. (**A**) Myricanol with 40 mg/kg; (**B**) myricanol with 20 mg/kg; (**C**) myricanol with 10 mg/kg; (**D**) vehicle control group; (**E**) tumor model group; and (**F**) isotype control. The yellow and brown particles represent positive VEGF expression; (**G**) Quantification of protein expressions in different groups by IHC.

We also examined tumor xenograft samples from each treatment group for the expressions of HIF-1α using IHC analysis. The relative expressions of HIF-1α did not change significantly in the myricanol and model groups compared with that in the vehicle group (*p* > 0.05, Figure 6).

**Figure 7:**
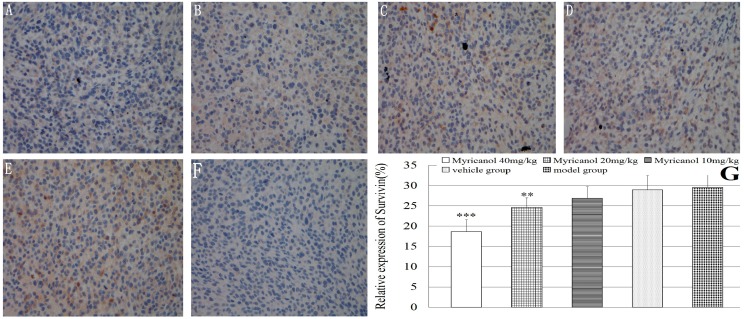
Immunohistochemical detection of survivin protein in A549 cells (magnification of 400×). The relative expression of survivin was determined by NIS-Elements D 3.2 image analysis system. (**A**) Myricanol with 40 mg/kg; (**B**) myricanol with 20 mg/kg; (**C**) myricanol with 10 mg/kg; (**D**) vehicle control group; (**E**) tumor model group; and (**F**) isotype control; (**G**) Quantification of protein expressions in different groups with IHC. ** *p* < 0.01, *** *p* < 0.001* vs.* vehicle group.

Finally, we examined tumor xenograft samples from each treatment group for the expressions of survivin using IHC analysis. The relative expressions of survivin were significantly lower in the high-dose and middle-dose myricanol groups than in the vehicle group (*p* < 0.01 to 0.001, Figure 7). The relative expression of survivin between the vehicle and model groups was not significant (*p* > 0.05). Myricanol can effectively inhibit the growth of human lung adenocarcinoma by inhibiting survivin expression.

Bax is also known as Bcl-2-like protein 4 or Bcl-2-associated X. Bax promotes apoptosis by antagonizing Bcl-2, which is specifically considered an important anti-apoptotic protein. Myricanol may increase the Bax/Bcl-2 ratio and eventually promote apoptosis. HIF-1α is a crucial activator responsible for lung cancer progression because it regulates the essential adaptive process for cancer cells to hypoxia. Furthermore, activated HIF-1α promotes the expression of VEGF and survivin, which subsequently benefits neovascularization and metastasis. Myricanol may regulate HIF-1α expression and affect VEGF and survivin expressions, thereby contributing to antitumor activity.

#### 2.1.3. Effects of Myricanol on the mRNA Expression of Apoptosis in A549 Cell Xenograft Model

**Figure 8:**
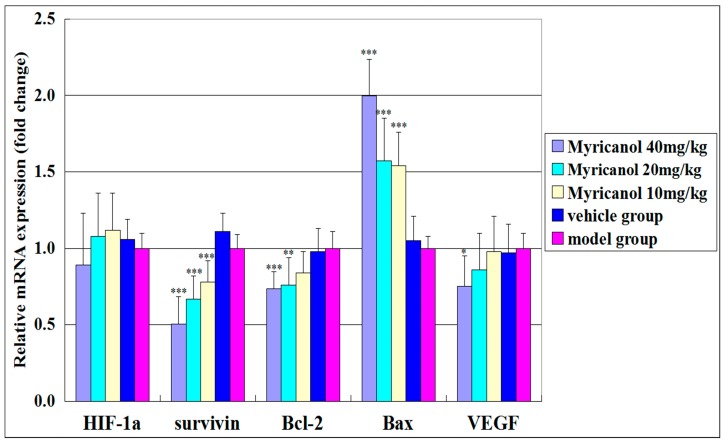
Effects of myricanol on the mRNA expressions of Bax, Bcl-2, VEGF, HIF-1α, and survivin in an A549 cell xenograft model. The mRNA expression of Bax significantly increased in a dose-dependent manner after myricanol treatment; the mRNA expressions of Bcl-2, VEGF, HIF-1α, and survivin were significantly down-regulated. These gene expression changes are implicated in the apoptotic pathway. Data are expressed as mean ± standard deviation, *n* = 8, * *p* < 0.05, ** *p* < 0.01, *** *p* < 0.001* vs.* vehicle control.

We used an A549 xenograft model to further study the antitumor effect of myricanol on the mRNA expression of apoptosis *in vivo*. The relative mRNA expression levels of Bax, Bcl-2, VEGF, HIF-1α, and survivin in tumor tissues were determined by quantitative real-time reverse transcriptase-polymerase chain reaction (qRT-PCR). Myricanol treatment significantly upregulated the mRNA expression of Bax and down-regulated the mRNA expressions of Bcl-2, VEGF, HIF-1α, and survivin in a dose-dependent manner compared with the vehicle group (*p* < 0.05 to 0.001, Figure 8). These results are consistent with those of IHC. These gene expression changes were associated with cell apoptosis. Myricanol may exert anti-tumor effect through the apoptosis pathway.

#### 2.1.4. Myricanol Induces Tumor Cell Apoptosis* in Vivo*

**Figure 9:**
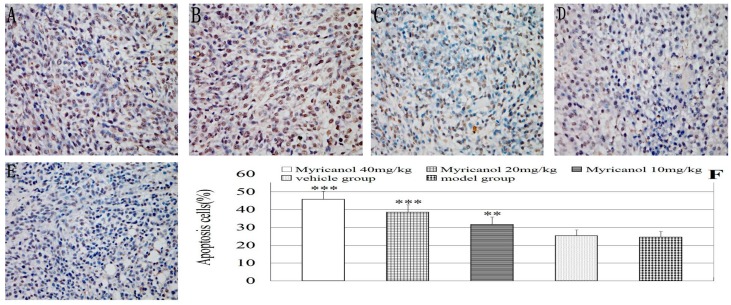
TUNEL staining in an A549 xenograft mouse model (magnification, ×400). (**A**) Myricanol with 40 mg/kg; (**B**) myricanol with 20 mg/kg; (**C**) myricanol with 10 mg/kg; (**D**) vehicle control group; and (**E**) tumor model group; (**F**) TUNEL performed to quantify the apoptotic A549 cells in different groups. The quantification of the apoptotic A549 cells were determined and shown in the diagram. The proportion of apoptotic-positive cells significantly increased in myricanol-treated cells compared with that in the vehicle control group. Data are expressed as mean ± standard deviation, *n* = 8, ** *p* < 0.01, *** *p* < 0.001* vs.* vehicle control. Brown-stained cell nucleus represents apoptotic cell; blue-stained cells represent normal A549 cells.

To determine if the administration of myricanol inhibits the growth of tumor xenografts by enhancing the apoptosis of the lung tumor cells *in vivo*, the xenograft tumors were subjected to TUNEL assay. The number of apoptotic-positive cells was counted in a high-power field (400× magnification). The proportion of apoptotic-positive cells in the myricanol-treated tumor tissues was significantly higher than that in the vehicle group (*p* < 0.01 to 0.001, Figure 9). The proportion of apoptotic-positive cells between the vehicle and model groups was not significant (*p* > 0.05). These data suggested that myricanol can significantly decelerate tumor growth *in vivo* by inducing apoptosis.

### 2.2. Discussion

In Asia, Chinese medicinal herbs have been widely used for centuries. *Myrica rubra* bark is an important medicinal plant in Asian countries because of its medicinal properties [11,12]. Previous pharmacological studies isolated many bioactive agents from *M. rubra* bark [13]. As a class of cyclic diarylheptanoids, myricanol is a bioactive agent extracted from *Myrica* bark [4]; this bioactive agent exhibits significant antitumor activity.

This study is the first to investigate and demonstrate the mechanism by which myricanol induces apoptotic cell death and antitumor activity in human lung adenocarcinoma A549 cells *in vivo*. The results suggested that myricanol can decrease the tumor weights of A549 cells *in vivo* by increasing apoptotic cells. Myricanol can also significantly upregulate the mRNA and protein expressions of Bax and downregulate the expressions of Bcl-2, VEGF, HIF-1α, and survivin in a dose-dependent manner. These data suggested that myricanol can significantly decelerate tumor growth *in vivo* by inducing apoptosis. Therefore, myricanol may be a clinical candidate to prevent and treat lung cancer.

Efficacy and specificity are necessary to provide successful cancer therapy. Apoptosis, or programmed cell death, is a normal physiological process that occurs during embryonic development and tissue homeostasis in adult animals [14]; apoptosis is a highly conserved eukaryotic cell suicide pattern. Cancer is a result of uncontrolled cell proliferation and apoptotic dysregulation [15]. Apoptosis comprises a series of typical morphological and biochemical events, including nuclear fragmentation, chromatin condensation, cell shrinkage, and rapid phagocytosis by neighboring cells [16]. Therefore, apoptotic induction is one of the effective approaches in antitumor therapy [17]. Our study suggested that myricanol effectively induces the apoptosis of A549 cells and may exhibit anticancer activities.

Bax, known as Bcl-2-associated X protein, is the first identified pro-apoptotic member of the Bcl-2 protein family [18]. Bcl-2 family members share one or more of the four characteristic domains of the Bcl-2 homology (BH), namely, BH1, BH2, BH3, and BH4, and can form heterodimers or homodimers [19]. Bcl-2 proteins act as anti- or pro-apoptotic regulators involved in various cellular activities. In healthy mammalian cells, Bax is mainly found in the cytosol; however, Bax is transferred to the mitochondrial outer membrane when apoptotic signaling is initiated, thereby inducing mitochondrial release of apoptotic factors and triggering apoptotic response [20,21]. Myricanol can up-regulate Bax and down-regulate anti-apoptotic Bcl-2 proteins in response to apoptosis *in vitro* [3]; this result is consistent with our data *in vivo*.

Hypoxia is a hallmark of many solid tumors; furthermore, hypoxia induces a series of changes in gene expression and participates in tumor progression [22]. HIFs are necessary to induce hypoxia-inducible gene expression in mammalian physiological and pathophysiological processes [23]. HIF-1 has a heterodimeric basic helix-loop-helix structure [24] composed of HIF-1α and an aryl hydrocarbon receptor nuclear translocator. HIF-1α-mediated hypoxia response is one of the most important transcription factors in target gene activation. HIF-1α induces various genes that are strongly associated with malignant alteration of tumors. HIF-1α also performs an important function in the prevention of hypoxia-induced apoptosis by up-regulating survivin and VEGF expressions [25,26].

HIF-1-induced cellular changes are important therapeutic targets of cancer therapy, particularly in therapy against refractory cancers. Therefore, targeting strategies are essential for cancer therapy to overcome HIF-1 active microenvironment. Myricanol may regulate HIF-1α expression and affect VEGF and survivin expressions, thereby contributing to antitumor activity.

In conclusion, myricanol could induce apoptosis in an A549 xenograft mouse model. Given that the highest dose of myricanol in this experiment was efficient in the treatment of A549 xenograft and that myricanol exhibited no obvious toxicity *in vivo*, we need to increase the doses of myricanol in future experiments. Considering that the solubility of myricanol in polyethylene glycol 400 was not good, modifying the myricanol structure is necessary to increase the solubility. This bioactive agent may be used as potential antitumor therapy for patients with NSCLC in the future. However, this agent requires further clinical trials for verification.

## 3. Experimental Section 

### 3.1. Reagents

Myricanol (98.2% purity, batch number: 20130822) was prepared as previously described [3]. In brief, dried *M. rubra* bark was ground into fine powder and sifted through a 20-mesh sieve. The powdered bark was extracted using ethanol, and the ethanol extracts were further extracted with chloroform. The chloroform extract was then subjected to column chromatography (silica gel layer column) separation with mobile phase elution (petroleum ether:ethyl acetate = 7:2). Myricanol content was detected by HPLC (Varian Prostar, Hanau, Germany). Chromatographic separation was performed using YMC-pack ODS-A column (250 mm × 4.6 mm × 5 μm). The myricanoll used in the experiments was dissolved at a concentration of 40 mg/mL in 100% polyethylene glycol 400 (PEG 400) as a high-dose group and diluted with PEG 400 before the experiment.

### 3.2. Animals and Cell Lines

Male BALB/c nude mice (five to six weeks old) were purchased from the SLRC Laboratory Animal Company (Shanghai, China). The mice were fed under humane and pathogen-free conditions. All of the animal experiments were conducted with the approval of the Animal Experimentation Ethics Committee of the Zhejiang Academy of TCM (Zhejiang, China).

Human lung carcinoma A549 cell line was obtained from the Integrated Traditional Chinese and Western Medicine Cancer Research Laboratory (Zhejiang Cancer Hospital, Zhejiang, China). The A549 cells were cultured in RPMI-1640 medium that contains 100 U/mL penicillin, 100 μg/mL streptomycin, and 10% heat-inactivated FBS at 37 °C in a humidified atmosphere of 5% CO_2_. Once 80% to 90% confluence was reached, the cells were trypsinized, harvested, and seeded into a new cell culture dish.

### 3.3. Tumor Model

Approximately 10^7^ A549 cancer cells were injected into the right axilla of the nude mice. Eight mice were housed per cage with free access to sterile water and standard laboratory chow diet. After 10 days, the diameter of the tumors reached 4 to 5 mm (approximately 100 mm^3^). The mice were randomized into five groups based on tumor size (*N* = 8): myricanol with three doses (40, 20, and 10 mg/kg body weight); polyethylene glycol 400 vehicle group (1 mL/kg); and tumor model group. All of the groups were treated with the corresponding drugs once daily by percutaneous intratumoral injection. The tumor volume was measured every other day in a matching cohort of mice using a Vernier caliper (Fisher, Pittsburgh, PA, USA) to measure maximal tumor diameter (L) and transverse diameter (W). The total tumor volume was calculated as (L × W^2^)/2. After 14 days, the mice were anesthetized with sodium pentobarbital and then sacrificed. Tumors were removed and weighed. The tumor tissues were collected in 10% neutral buffered formalin or liquid nitrogen for use. TIR was calculated as follows: TIR = (average tumor weight of control group − average tumor weight of administration group)/average tumor weight of control group × 100%.

### 3.4. Immunohistochemical

Excised tumor specimens were fixed in 10% neutral buffered formalin for 24 h. The tissues were embedded in paraffin, and sections with 3 μm thickness were sliced. The sections were deparaffinized and rehydrated. Afterward, the sections were dewaxed by xylene and processed by graded ethanol debenzolization. After retrieving the heat-induced antigen, the sections were allowed to cool for 30 min and then rinsed in distilled water. Endogenous peroxidase was inhibited by incubating the tissue sections with 3% hydrogen peroxidase for 15 min at room temperature. Specific epitope binding was then blocked by incubation for 40 min with 20% goat serum. Slides were incubated at 4 °C overnight with dilutions of primary antibodies against Bax (dilution 1:200), Bcl-2 (dilution 1:200), VEGF (dilution 1:100), HIF-1α (dilution 1:100), and survivin (dilution 1:100). An isotype control was also employed to identify non-specific binding from the secondary antibody. These primary antibodies were all purchased from Epitomics (Burlingame, CA, USA). The samples were subsequently incubated with the secondary antibodies for 30 min. Slides were then visualized using 3,3'-diaminobenzidine (DAB) chromogen (LabVision Corp., Fremont, CA, USA) and counterstained with hematoxylin and eosin. The sections were washed in PBS, dried, mounted in neutral gum, and observed at 400× magnification using an NIS-Elements D 3.2 image analysis system. Five no-repeat high-power field images were randomly selected. Brown particles were considered as positive area; protein expressions in different groups were quantified and evaluated.

### 3.5. Total RNA Isolation and Quantitative Real-Time PCR Analysis

The total RNA of each tumor specimen was isolated using an E.Z.N.A total RNA kit (Omega Bio-tech Inc., Norcross, GA, USA) in accordance with the manufacturer’s protocol. Total RNA samples were suspended in diethylpyrocarbonate-treated water. The purity and concentration of the RNA samples were determined using a NanoDrop spectrophotometer (ND-2000, NanoDrop Technologies, Waltham, MA, USA). To synthesize cDNA, 1 μg of total RNA was added in a 25 μL reaction volume using a first-strand cDNA synthesis kit (GeneCopoeia, Rockville, MD, USA). The reaction products of reverse transcription were maintained at −20 °C until use.

All of the oligonucleotide primers were designed using PerlPrimer software and synthesized commercially (Sangon Biotechnology, Shanghai, China). The sequences of the primers are as follows: 5'-GGTCTCCTCTGACTTCAACA-3' (forward) and 5'-AGCCAAATTCGTTGTCATAC-3' (reverse) for GAPDH, 5'-AAGCTGAGCGAG TGTCTCAAG-3' (forward) and 5'-CAAAGTAGAAAAGGGCGACAAC-3' (reverse) for Bax,5'-ATGTGTGTGGAGAGCGTCAAC-3' (forward) and 5'-AGAGACAGCCAGGAGAAATCAAAC-3' (reverse) for Bcl-2, 5'-TGACGGACAGACAGACAGACACC-3' (forward) and 5'-AGAACAGCCCAGAAGTTGGACGA-3' (reverse) for VEGF, 5'-AGCCAGACGATCATGCAGCTACTA-3' (forward) and 5'-TGTGGTAAT CCACTTTCATCCATTG-3' (reverse) for HIF-1α, and 5'-AGGTCATCTCGGCTGTTCC TG-3' (forward) and 5'-TCATCCTCACTGCGGCTGTC-3' (reverse) for survivin. qRT-PCR was performed on 7500 StepOnePlus™ (Applied Biosystems, Foster, CA, USA) for 40 cycles using SYBR premix EX *Taq* (TaKaRa, Dalian, China) in accordance with the manufacturer’s protocol. The reactions were conducted in duplicate in a 25 μL reaction volume in a 96-well plate. The reaction mixtures without cDNA served as negative controls. Two-step PCR conditions were employed as follows: initial denaturation at 95 °C for 30 s; 40 cycles of denaturation at 95 °C for 5 s; annealing and extension at 60 °C for 30 s; confirmation with melting curve analysis at 95 °C for 15 s; and 50 and 95 °C for 15 s. GAPDH was used as a housekeeping gene to normalize the expression of the target genes (Bax, Bcl-2, VEGF, HIF-1α, and survivin) by using 2^−ΔΔ*C*t^, where ΔΔ*C*_t_ = Δ*C*_t_ (drug treatment) − Δ*C*_t_ (background), Δ*C*_t_ = *C*_t_ (target gene) − *C*_t_ (GAPDH), and *C*_t_ is the threshold cycle.

### 3.6. Terminal Deoxynucleotidyl Transferase dUTP Nick End Labeling (TUNEL) Assay

TUNEL assay is a common method used to detect DNA fragmentation resulting from apoptosis. In this assay, the presence of nicks in the DNA is identified by terminal deoxynucleotidyl transferase (TdT), an enzyme that catalyzes the addition of dUTPs secondarily labeled with a marker. TdT may also be used to label cells with severe DNA damage. TUNEL assay was performed in accordance with the manufacturer’s instructions (Beyotime Institute of Biotechnology, Beijing, China). In brief, the tumor tissues were fixed with 10% formalin for 4 h and then embedded in paraffin. Slices were deparaffinized and pretreated with proteinase K (20 mg/mL; Millipore, Boston, MA, USA) for 15 min. The samples were then placed in 3% H_2_O_2_ for 10 min at room temperature. The tumor sections were subsequently incubated with TdT enzyme and bound to anti-digoxigenin conjugate. The slides were stained with DAB peroxidase substrate, counterstained with 0.5% methyl green for 10 min, and mounted under coverslips. The slides were examined under a light microscope, and images of five random fields of view per section at 400× magnification were captured. The percentage of apoptotic cells was scored as an average of the ratio of TUNEL-positive cells to the total number of cells present in each field.

### 3.7. Statistical Analysis

One-way ANOVA was performed. The quantitative values were expressed as mean ± standard deviation, and the differences between means were statistically analyzed by SPSS 19.0 (SPSS Inc., Chicago, IL, USA) and by multiple comparison test. Asterisks indicated statistical significance as follows: * *p* < 0.05; ** *p* < 0.01; *** *p* < 0.001. Controls of statistical analysis were specified in each figure. All of the experiments were performed at least three separate times.

## 4. Conclusions

Myricanol can significantly induce inhibitory effects on xenografts of lung adenocarcinoma A549. This mechanism can be associated with apoptotic cell death by up-regulating Bax expression and down-regulating Bcl-2, VEGF, HIF-1α, and survivin expressions. Therefore, myricanol may be a clinical candidate to prevent and treat lung cancer.
